# Thermal treatment using microwave irradiation for the phytosanitation of *Xylella fastidiosa* in pecan graftwood

**DOI:** 10.1371/journal.pone.0244758

**Published:** 2021-01-20

**Authors:** Angelyn Hilton, Myunghwan Jeong, Jui-Hung Hsu, Fan Cao, Woongchul Choi, Xinwang Wang, Choongho Yu, Young-Ki Jo

**Affiliations:** 1 Department of Plant Pathology and Microbiology, Texas A&M University, College Station, Texas, United States of America; 2 USDA-ARS Pecan Breeding and Genetics, Somerville, Texas, United States of America; 3 Department of Mechanical Engineering, Materials Science and Engineering, Texas A&M University, College Station, Texas, United States of America; Auburn University, UNITED STATES

## Abstract

Pecan bacterial leaf scorch caused by *Xylella fastidiosa* is an emerging disease for the U.S. and international pecan industries and can be transmitted from scion to rootstock via grafting. With the expanse of global transportation and trade networks, phytosanitation is critical for reducing the spread of economically significant pathogens, such as *X*. *fastidiosa*. We developed and evaluated thermal treatments using microwave irradiation and microwave absorbers [sterile deionized water (dH_2_O) and carbon nanotubes (CNTs)] as novel disinfectant methods for remediating *X*. *fastidiosa* in pecan scions. Partial submergence of scions in dH_2_O or CNT dispersions resulted in the transport of microwave absorbers in the xylem tissue via transpiration but did not compromise plant health. The microwave absorbers effectively transferred heat to the scion wood to reach an average temperature range of 55–65°C. Microwave radiation exposure for 6 sec (3 sec for two iterations) of CNT- or dH_2_O-treated scions reduced the frequency of *X*. *fastidiosa*-positive in pecan scions without negatively affecting plant viability when compared to the control group (dH_2_O-treated with no microwave). The efficacy of the new thermal treatments based on microwave irradiation was comparable to the conventional hot-water treatment (HWT) method, in which scions were submerged in 46°C water for 30 min. Microwave irradiation can be employed to treat *X*. *fastidiosa*-infected scions where the conventional HWT treatment is not feasible. This study is the first report to demonstrate novel thermal treatment methods based on the microwave irradiation and microwave absorbers of dH_2_O and CNT as an application for the phytosanitation of xylem-inhabiting bacteria in graftwood.

## Introduction

Phytosanitation involves the remediation of quarantined plant pathogens and insect pests from distributed plant germplasm. The Food and Agriculture Organization of the United Nations (FAO) estimates that plant diseases account for more than $200 billion in losses to the global economy (fao.org). With current trends in globalization, it is critical to screen and remediate infected plant germplasm before national and international transport to restrict the spread of potentially dangerous pathogens [1]. The International Plant Protection Convention, governed by the Commission on Phytosanitary Measures (CPM), has adopted and implemented over 100 international standards for the diagnostics and phytosanitation of six major pests, including *Xylella fastidiosa*. *X*. *fastidiosa* is a fastidious, gram-negative, rod-shaped bacterium known to infect between 343 to 595 different plant species [2]. Significant yield reductions caused by *X*. *fastidiosa* have been recorded in host plants, such as grape and citrus, and can result in industry losses over $100 million annually [3].

**Fig 1:**
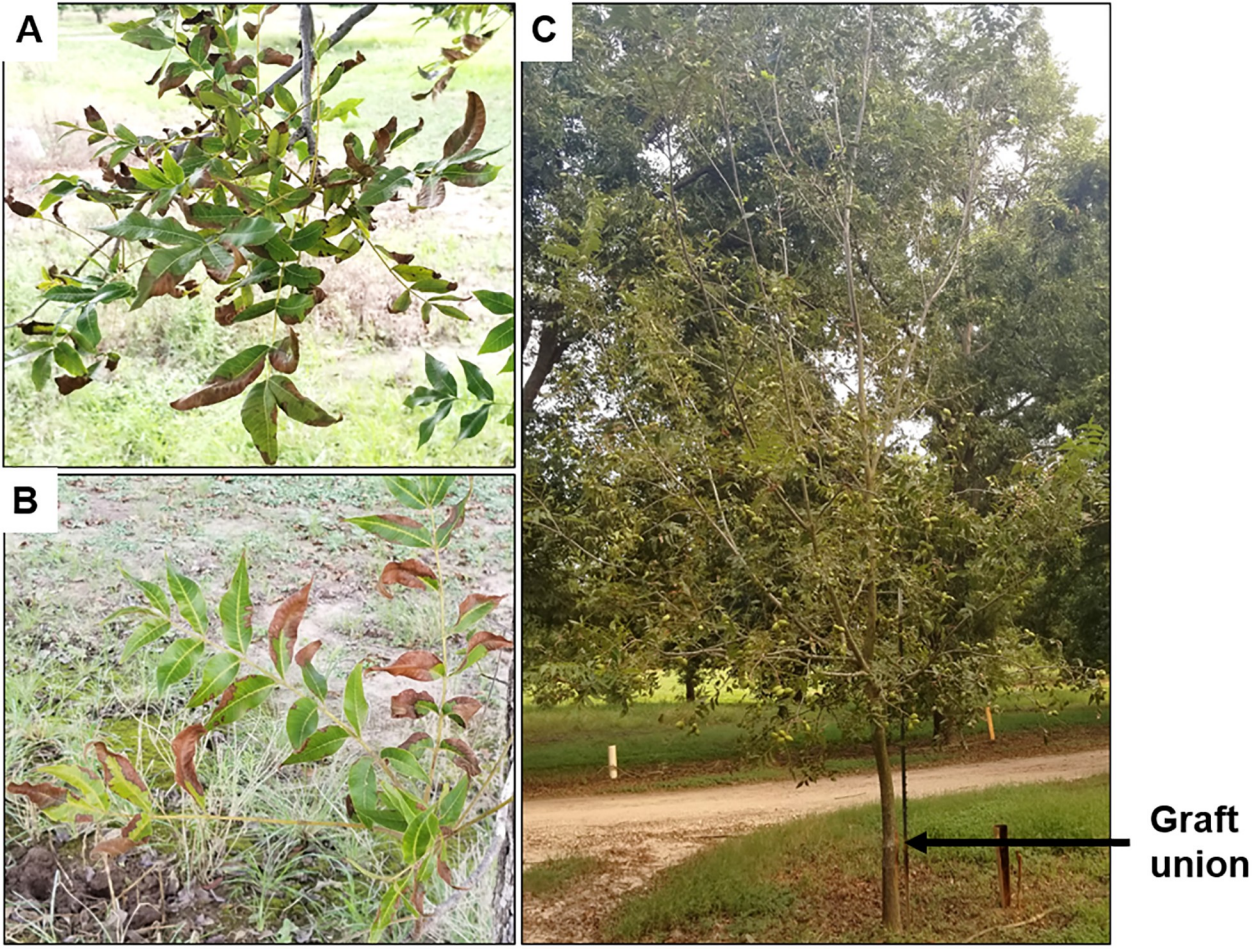
Symptoms of pecan bacterial leaf scorch (PBLS) caused by *Xylella fastidiosa* in pecan. (A) Foliar symptoms of PBLS on leaves of the scion (*i*.*e*., in the canopy above the graft union). (B) PBLS symptoms on leaves of the rootstock (*i*.*e*., on the lower half below the graft union). (C) Premature defoliation of the canopy due to PBLS. Arrow indicates the graft union.

Pecan bacterial leaf scorch (PBLS), caused by *X*. *fastidiosa*, is an emerging disease of pecan (*Carya illinoinensis*) and related species of *Carya* across the southern U.S. [4–7]. In pecan, *X*. *fastidiosa* causes foliar disease symptoms as light-brown necrotic lesions that first develops on leaflet tips and margins and later progresses toward the leafstalk or petiole, followed by leaflet abscission (Fig 1). Xylem occlusion caused by the bacterium can also negatively influence kernel development, which has a direct impact on nut quality and yield [8]. Researchers estimate that the economic significance of PBLS reductions in nut yield by up to 12%, could result in value losses over $450/ha.

*X*. *fastidiosa* is xylem limited and can be transmitted in pecan via grafting. Grafting is a method of joining different plant varieties by fusing a scion (upper part of the plant) with a rootstock (lower part of the plant) to grow as a single plant [9–12]. Sanderlin and Melanson [10] found an average transmission rate of *X*. *fastidiosa* from infected scions to clean rootstocks to be 21.3%. This is a critical issue as vegetative propagation of pecan is predominantly performed through grafting. Currently, hot-water treatment (HWT) is the only proposed phytosanitary method to eliminate *X*. *fastidiosa* from pecan scion wood [10, 13]. The establishment of additional novel and effective phytosanitary methods for PBLS and other diseases caused by *X*. *fastidiosa* is vital to sustaining both commercial and federal interests in disease-free germplasm.

The utilization of nano-sized materials, such as carbon nanotubes (CNTs), is an emerging research topic with an increasing array of potential applications throughout multiple disciplines [14]. CNTs are one-dimensional carbon structures with diameters in the nanometer scale and an aspect ratio of higher than 1,000. Single (single-walled CNT, SWCNT) or multiple (multi-walled CNT, MWCNT) graphite layers are rolled up to form cylindrical structures whose diameter ranges from a few to over 100 nanometers [15–17]. CNTs have been used as transporters in biotechnology applications, which can permeate tumor cells, bacteria, plant cells, and animal tissues, to deliver drugs, DNA, proteins, or physical cancer therapy [14, 18, 19]. More recently, scientists have gained greater insight into the impacts of CNTs on crop production and have proposed the use of CNTs to increase overall biomass and yield [20, 21].

CNTs have unique mechanical, thermal, and electrical properties, which can be used as a means to alter the functionalization of chemical and biological structures within a reactive environment [14, 22, 23]. For example, free electrons can move across CNT layers at a rate of approximately one per carbon atom and enable CNTs to absorb electromagnetic radiation in the microwave frequency range [24]. Microwave absorption allows CNTs to transport energy in the form of heat to neighboring structures by irradiation, rather than conduction and convection, which are the dominant processes for delivering energy in traditional heating methods [25, 26]. Heating by microwave irradiation has several advantages, including lower energy consumption, faster processing than conduction and convection, and capabilities for selective heating [25]. Microwave irradiation has been used to pasteurize contaminated food crops, eliminate potential phytopathogens from infected seed, soils, or agricultural equipment, and to control weeds and insect pests [27–29]. However, there are no reports on the use of microwave irradiation for the treatment of plant graftwood or the remediation of *X*. *fastidiosa* from infected hosts. In this study, we aim to develop novel phytosanitary thermal treatments that utilize water, CNTs and microwave irradiation to reduce or eliminate *X*. *fastidiosa* from pecan scion wood.

## Materials and methods

### Harvesting of pecan scion wood

Scion wood was harvested from the PBLS-susceptible pecan cultivar, ‘Cape Fear’ (CSV 18–11), at the USDA-ARS Pecan Breeding and Genetics Program in Burleson County, TX. The tree had exhibited moderate to severe symptoms of PBLS and *X*. *fastidiosa* had been detected by polymerase chain reaction (PCR) and enzyme-linked immunosorbent assay (ELISA) in current and previous growing seasons. Samples were harvested in late January and early February of 2018 and 2019, respectively, during the dormant phase of the seasonal lifecycle of pecan. Pruners sanitized with 70% ethanol were used to excise dormant, 1-year-old scion wood from the mid-canopy throughout each of the directional quadrants: north, south, east and west. The scions were between 0.5 and 1.3 cm in diameter and cut with pruners to 15 cm in length. The average mass of scions was 3.2 g ± 0.1. Scions were randomly grouped and wrapped in a moist paper towel to prevent excess water loss, sealed in plastic bags, and stored at 4°C for up to 4 months.

### Microwave absorbers

Sterile deionized water (dH_2_O), commercial P2 single-wall CNTs (SWCNTs) (#P2-SWNT, Carbon Solution, Inc., Riverside, CA) or multi-wall CNTs (MWCNTs) (#030103, Cheap Tubes, Inc., Grafton, VT) were used as microwave absorbers. The SWCNTs had a carbonaceous purity of over 90% and without functionalities. The bundle length and diameter were 0.5–1.5 μm and 4–5 nm, respectively. The MWCNTs were purified by concentrated acid chemistry and produced using the catalytic chemical vapor deposition (CCVD) method [30]. The outer diameter, inside diameter, ash, purity, length, specific surface area, bulk density, and true density were 8–15 nm, 3–5 nm, < 1.5%, > 95%, 10–50 μm, 233 m^2^/g, 0.15 g/cm^3^ and ~2.1 g/cm^3^, respectively. The important properties including electrical conductivity of CNTs used in this study were well characterized [16, 24, 31–36].

A CNT dispersion was prepared at a density of 0.1% (w/v) by dispersing CNTs with soluble starch (#AAA11961-36, VWR, Radnor, PA) in sterile dH_2_O using a probe-type ultrasonic homogenizer (100 W, XL2000, Misonix Inc.). Visual observations indicated the CNT dispersion had maintained stability for approximately 1 week without precipitation. Further dilutions of the stock 0.1% (w/v) CNT dispersion in sterile dH_2_O were prepared before optimization and analysis of CNT treatments on scions. The probe-type ultrasonic homogenizer was used to disperse the CNTs in solution before all scion treatments to ensure uniform distribution and prevent aggregation of the CNTs. The proximal end of each scion was pruned by approximately 2 cm to remove any dead plant tissue and submerged into 5 mL of CNT dispersion or sterile dH_2_O. The treated scions were incubated in the dark for seven days at 8°C to maintain dormancy of the buds. Following incubation, samples were stored in sealed plastic bags for 1–2 days at 4°C prior to microwave exposure.

To verify the infiltration of CNTs into the plant xylem, scions were first treated with 0.1% (w/v) SWCNT dispersion or sterile dH_2_O, as previously described. Lateral cross-sections, 3–5 mm in width, were dissected from CNT- and dH_2_O-treated scions using pruners sanitized with 70% ethanol, to prevent cross-contamination. Three cross-sections were dissected approximately 3–4 cm from the distal and proximal ends, as well as from the central section of the scion wood. The dissected samples were placed on glass slides and observed under a stereoscope at 40X magnification. A DinoCapture 2.0 (AnMo Electronics Corporation, New Taipei City, Taiwan) camera was used to capture images for comparison.

### Optimization of microwave irradiation treatments

SWCNTs and MWCNTs were compared to determine the optimum structure and density for future scion treatments. SWCNT and MWCNT dispersions were first diluted to 0.01%, 0.001%, and 0.0001% (w/v) in sterile dH_2_O. The diluted CNT dispersions were used to treat scion samples, as previously described. A subset of scions was also treated with sterile dH_2_O, which served as a control group for comparison. Six scion replicates were tested per treatment group. Following CNT or dH_2_O treatment, a hole was drilled inside the pith along the longitudinal plane of each scion at approximately 6–7 cm in length. A 700-Watt commercial microwave oven (Hamilton Beach 0.7 cu ft) was used to expose the CNT- or dH_2_O-treated scions to electromagnetic radiation at a frequency of 2450 MHz. The scions were placed on the rotary plate and microwaved for 4 sec. Immediately following the microwave treatment, a T-type thermocouple was inserted into the drill hole of the scion to obtain a single-point temperature measurement. The CNT type and density with the highest recorded temperature difference from the dH_2_O-treated control group were used for future experimentation.

The T-type thermocouple and a thermal infrared camera (FLIR ONE Pro, Official FLIR store, Wilsonville, OR) were compared to determine the differences between temperature measurements of the two instruments. Scions were individually placed on the rotary plate and microwaved for 5 sec. Immediately following the microwave treatment, a thermocouple was inserted into the scion drill hole to obtain a single-point temperature measurement, as previously described. Three-point temperature measurements were taken using the thermal camera immediately following the temperature estimate when using the thermocouple. Three scion sample replicates were tested by the thermocouple and thermal camera to make comparisons.

The testing of microwave exposure times was conducted to optimize standard operating procedures. Previous research demonstrates an effective temperature range of 45 to 55°C to eliminate *X*. *fastidiosa*, which served as a target for the optimization of microwave irradiation treatments [10]. Scion samples were first treated with dH_2_O, 0.01% MWCNT (w/v), or 0.1% MWCNT (w/v) dispersion, as previously described, and then dried for two days at room temperature (R.T., ~ 25°C). Previous comparisons between the thermal camera and thermocouple revealed a lack of uniform heat distribution, indicating indirect exposure of the microwave radiation. Therefore, the microwave was placed on its side, and the dH_2_O- and CNT-treated scions were fixed laterally on a mount, 267 mm in height, and 90 mm below the cavity magnetron. Scions were microwaved individually for 3 sec or 5 sec, and to further improve the uniformity of heat distribution, CNT- or dH_2_O-treated scions were also microwaved and then adjusted in iterations by moving the scion laterally on the mount for more direct exposure to the cavity magnetron. In the first iteration, the distal and central sections of the scion were placed on the center of the mount within the direct focus area of the cavity magnetron. The scion was then readjusted in the second iteration so that the proximal and central sections were then directly exposed to microwave radiation within the focus area. For microwave treatments consisting of more than two iterations, scions were adjusted by alternating between the distal and proximal end for each iteration. In addition to microwave treatments of 3 sec or 5 sec, three other treatments were included in the analysis in which scions were microwaved for 2 sec per scion readjustment in six iterations (12 sec total), 3 sec in two iterations (6 sec total), or for 3 sec in three iterations (9 sec total). The resulting temperature change was estimated using six-point measurements generated from the thermal camera immediately (within 3 sec) following the last iteration. A total of six replicates per treatment were used for optimization and analysis. Significant differences between grouped comparisons of scion treatments were determined by analysis of variance (ANOVA), followed by a post-hoc Tukey’s test of mean separation (*P* < 0.05) using SAS V9.4 M6 University Edition.

The previous experiment was repeated with microwave exposure treatments of 3 sec or 3 sec with scion readjustment in two iterations (6 sec) on CNT- or dH_2_O-treated scions. Scions were first treated with 0.01% (w/v) MWCNT dispersion or sterile dH_2_O and then exposed to microwave radiation on a mount with direct exposure to the cavity magnetron, as previously described. In this latter experiment, temperature readings of the scions were determined from three-point measurements taken using the thermal camera. The distribution of measurements was compared to determine the optimum microwave treatment based on the effective temperature range. A total of six scion replicates were included for each treatment condition. Significant differences between grouped comparisons of scion treatments were determined by ANOVA, followed by a post-hoc Tukey’s test of mean separation *(P <* 0.05), as previously described.

### Scion viability assay

Scion wood viability was determined following thermal treatments combining dH_2_O or 0.01% (w/v) MWCNTs with microwave irradiation for 6 sec (3 sec for two iterations following scion readjustment). Two control groups were also included in which scions were treated with dH_2_O or 0.01% (w/v) MWCNTs and no microwave irradiation. The treated scions were fitted onto an aeroponic system (Clone King Aeroponic Systems, Albuquerque, NM) in a serpentine pattern and completely randomized design (CRD) (S1 Fig). Scion samples are inserted into foam grommets affixed to a lid. The bucket frame, which is outfitted with a submersible recirculating pump and mist spray system, was filled with 15 L of dH_2_O supplemented with 1 ppm azoxystrobin (Heritage TL, Syngenta, Greensboro, NC) to limit fungal contamination, and changed after seven days. The treated scions were incubated under a 16 hr/8 hr light/dark cycle at R.T. for a total of 14 days and monitored for the outgrowth of axillary buds or bud-break. A total of 16 replicates per treatment were tested. As a function of plant viability, a chi-square analysis was performed using SAS V9.4 M6 University Edition to determine any significant differences (*P* < 0.05) based on the proportion of scions that initiated outgrowth from axillary buds or underwent bud-break per the total number of samples tested for each treatment.

### Presence/absence determinations of *X*. *fastidiosa* in scions following thermal treatments

The presence of *X*. *fastidiosa* was evaluated after the following two thermal treatments: 1) 0.01% (w/v) MWCNT dispersion and 6 sec microwave irradiation (3 sec for two iterations following scion readjustment), and 2) dH_2_O and 6 sec microwave irradiation (3 sec for two iterations following scion readjustment). As an industry standard, hot-water treatment (HWT) [10] was also included. For HWT, scions were completely submerged in a 46°C water bath for 30 min. To establish a baseline, two negative control groups were included, dH_2_O or MWCNT treatment without microwave irradiation. The experiment was conducted three times. A total of 16 replicates (scions) per treatment were tested, consisting of six replicates in the first experiment and five replicates in the second and third experiments. Following the treatments, scion samples were incubated on the aeroponic system in a CRD for two weeks, as previously described.

After the incubation period, a 10 mg sample was taken from the central section of the scions, frozen in liquid nitrogen, and stored at -80°C prior to DNA extraction. Total gDNA was isolated from scion tissue samples using the DNeasy Plant Mini Kit (#69104, Qiagen, Hilden, Germany) based on the manufacturer’s protocols. The DNA pellets were eluted in 50 μL nuclease-free dH_2_O, and the concentration was estimated using a NanoDrop 2000 ultraviolet (U.V.)-Vis spectrophotometer (ThermoFisher Scientific, Waltham, MA). The DNA concentration was adjusted to 10 ng/μL prior to the downstream analysis.

The previously reported probes and primer sets specific to *X*. *fastidiosa* subsp. *multiplex* HL (hypothetical protein) region and the actin-encoding gene of pecan [37, 38] were used for qPCR amplification (Table 1). The HL primers and probe were designed specifically from *X*. *fastidiosa* strain sequences obtained from PBLS-infected pecan trees, and will not bind to the DNA of other biological contaminants [37]. The amplification of the actin loci of pecan served as the internal positive control (IPC) to verify the efficacy of the qPCR reaction and the lack of inhibitors that may interfere with DNA amplification. All qPCR reactions were prepared on optical 96-well plates using the TaqPath ProAmp Master Mix (#A30871, ThermoFisher Scientific, Waltham, MA) as chemistry. The qPCR profile using standard cycling was performed based on manufacturer’s protocols specific for plant genotyping. Fluorescent intensities in each reaction well were determined by an Applied Biosystems StepOne Real-Time PCR system (ThermoFisher Scientific). Data acquisition and analysis were performed using the StepOne Software v2.3 with default call settings. Determination of ΔRn, the signal strength generated in the qPCR reaction, was calculated by the StepOne Software v2.3 as the level of fluorescence above the baseline and within the exponential growth region.

**Table 1:** Primer sets and probes for *Xylella fastidiosa* HL and pecan actin used for qPCR reactions.

Primer set	Tm (°C)	Forward and reverse sequence (5'-3')
HL Probe [37]	59.4	/56-FAM/TTGCTGCGA/ZEN/ATATCTTCCACGGTC/3IABkFQ/
HL4 [37]	53.5 54.1	GTGAAACATGCTGCCGAT GCAGGCAGCAACGATAC
Actin Probe [37, 38]	61.1	/5HEX/TGGAAGAGA/ZEN/ACTTCTGGGCAACGG/3lABkFQ/
Actin [37, 38]	54.6 54.7	TTGTATGTGGTCTCGTGGATTC ATCACAATTGGAGCTGAGAGG

A final gDNA concentration of 1 ng/μL was used as the template for each unknown sample reaction, as well as six non-template controls consisting of nuclease-free dH_2_O as the template. A positive control of purified *X*. *fastidiosa* subsp. *multiplex* gDNA was serially diluted at 1, 0.2, 0.04, 0.008, and 0.002 ng per 10 ng/μL reaction to create a standard curve. The standard curve was analyzed to determine qPCR efficiency. Each sample was tested in triplicate, and consistency in amplification was required for *Xylella*-positive determinations with a threshold cycle (Ct) cutoff value = 38. A chi-square analysis was performed using SAS V9.4 M6 University Edition to determine the thermal treatment effect on the proportion of scions that tested positive for *X*. *fastidiosa* in comparison to the nontreated control group (*P* < 0.05).

## Results

### Microwave absorbers

**Fig 2:**
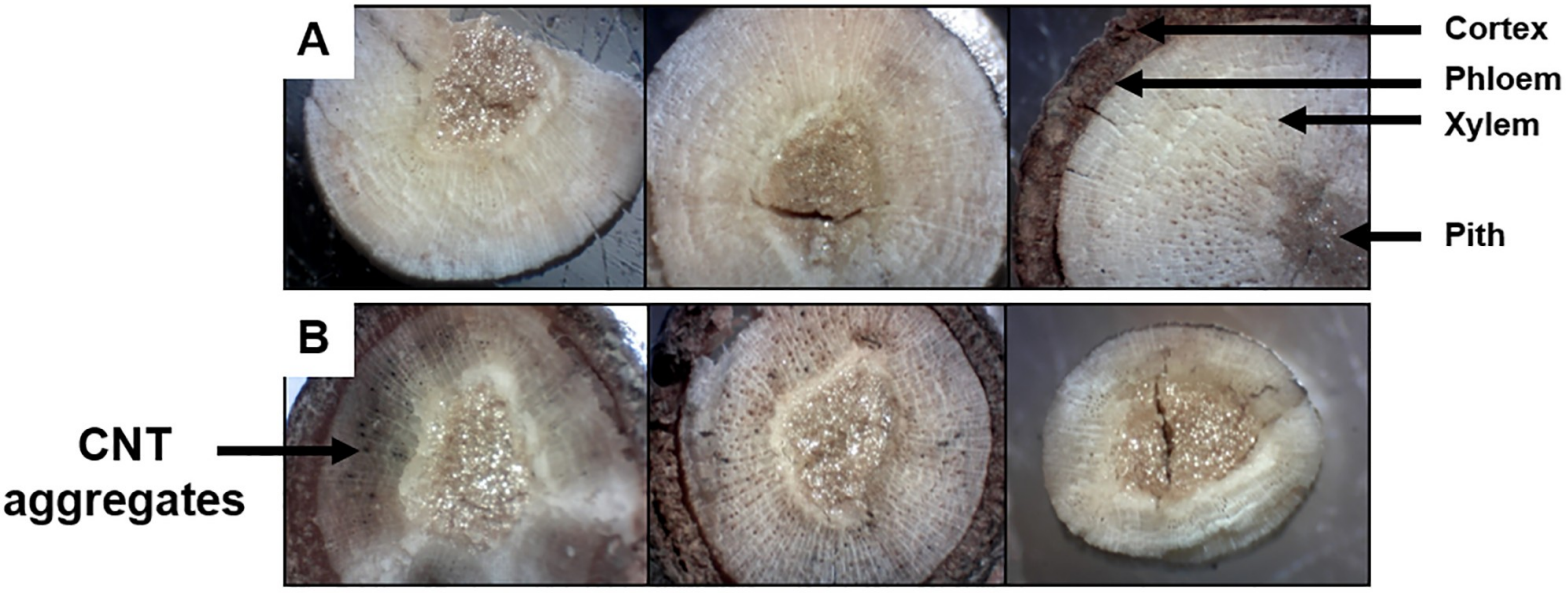
Microscopic inspection of carbon nanotube (CNT) transpiration in the xylem. From left to right are subsamples dissected from the proximal, central, and distal sections of the wood, respectively. (A) Scion samples treated with sterile deionized water (dH_2_O). Arrows identify the anatomical features of a transect of the scion. (B) CNT-treated scion samples. Arrow indicates the presence of aggregated CNT particulates.

Dissected cross-sections of the proximal, central, and distal ends of the SWCNT- or dH_2_O-treated scion samples were compared under a stereoscope at 40X magnification (Fig 2). In SWCNT-treated scions, observations of cross-sections revealed that CNTs are transpired through the xylem. The xylem tissue appeared darkened, and aggregated CNTs were identified along the periphery of the vessel elements in SWCNT-treated scions. Conversely, in dH_2_O-treated samples, the xylem tissue was lighter in color and free of particulates around vessel elements. It was evident that higher densities of the CNT aggregates were present in the proximal end, and then steadily decreased towards the central section and distal end of the scion. Following the one-week treatment with microwave absorbers, scions were observed to absorb 1–2 mL of dH_2_O or CNT dispersion. Considering the starting density of the CNT dispersion as 0.001 g/mL (0.1% [w/v]), it was estimated that maximum amount of absorbed CNTs was 0.002 g per treated scion.

SWCNTs and MWCNTs with densities of 0.01%, 0.001%, and 0.0001% (w/v) were compared to optimize future scion treatments with microwave absorbers. The 0.01% MWCNT treatment resulted in the highest temperature increase following microwave exposure when compared to dH_2_O, SWCNT treatments, or MWCNT treatments at lower densities (S2 Fig). Therefore, 0.1% (w/v) or 0.01% (w/v) MWCNT treatments were used for subsequent experiments.

### Optimization of microwave irradiation treatments

**Fig 3:**
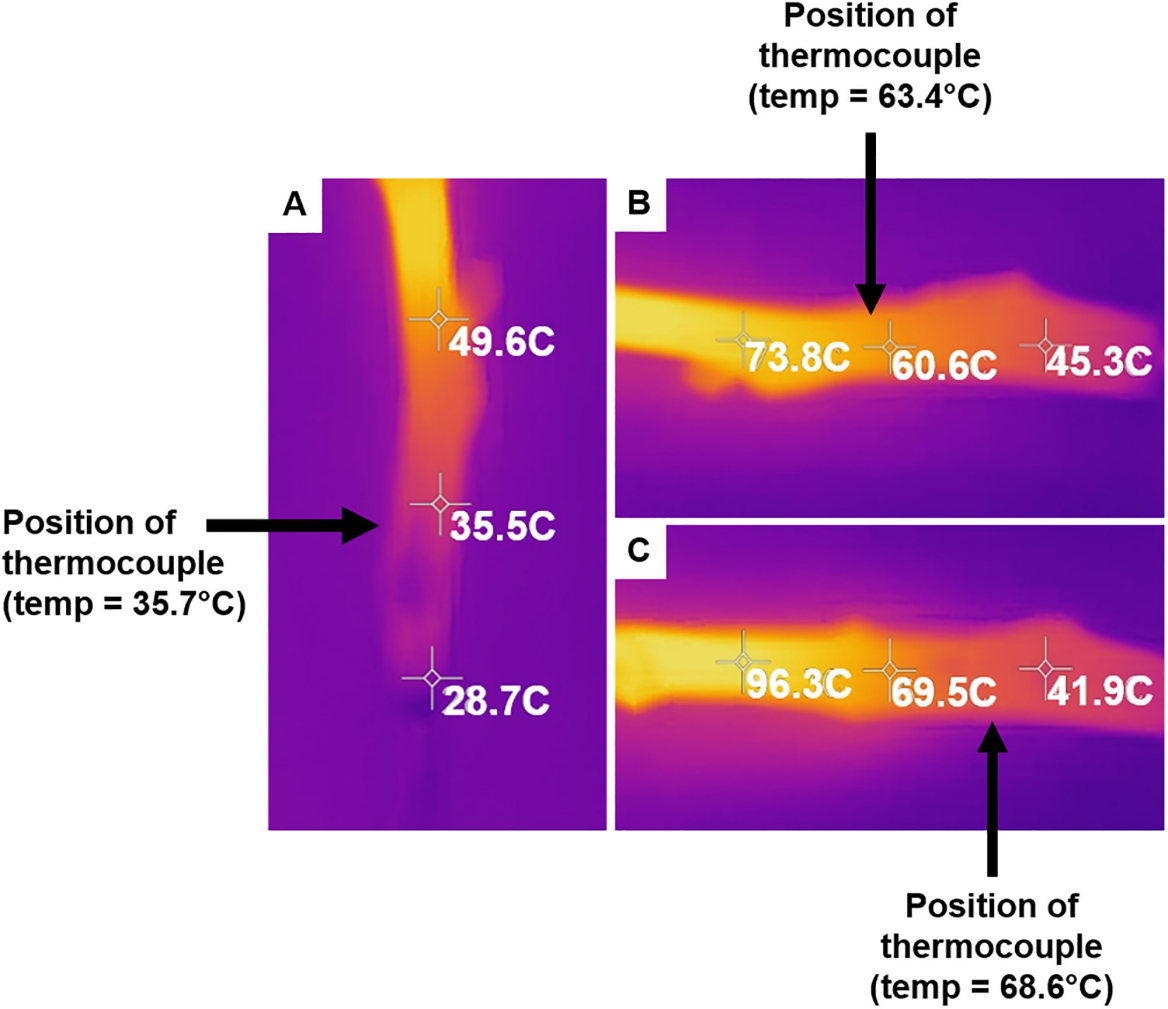
Comparison of temperature measured with a thermal camera and a thermocouple. Three-point measurements were taken with the thermal camera of pecan scion replicates following carbon nanotube (CNT) treatment. A hole was drilled laterally inside the scion pith, and a thermocouple was inserted for single-point temperature measurements. Arrows indicate the position of the thermocouple upon measurement. (A) The thermocouple measured temperature = 35.7°C. (B) The thermocouple measured temperature = 63.4°C. (C) The thermocouple measured temperature = 68.6°C.

Two different tools, a thermocouple and thermal camera, were used to estimate the temperature change of scions following microwave radiation exposure and compared to validate accurate internal temperature measurements (Fig 3). The temperatures measured using the thermocouple were comparable to the temperatures estimated by the thermal camera, dependent on the site of the point-measurement by the thermocouple when inserted into the scion. The thermal camera revealed a range of temperature changes that were not well distributed across the scion. Due to increased temperature variability from a lack of uniform heating, the thermal camera was used in subsequent experiments to estimate the temperature following microwave irradiation of scions.

**Fig 4:**
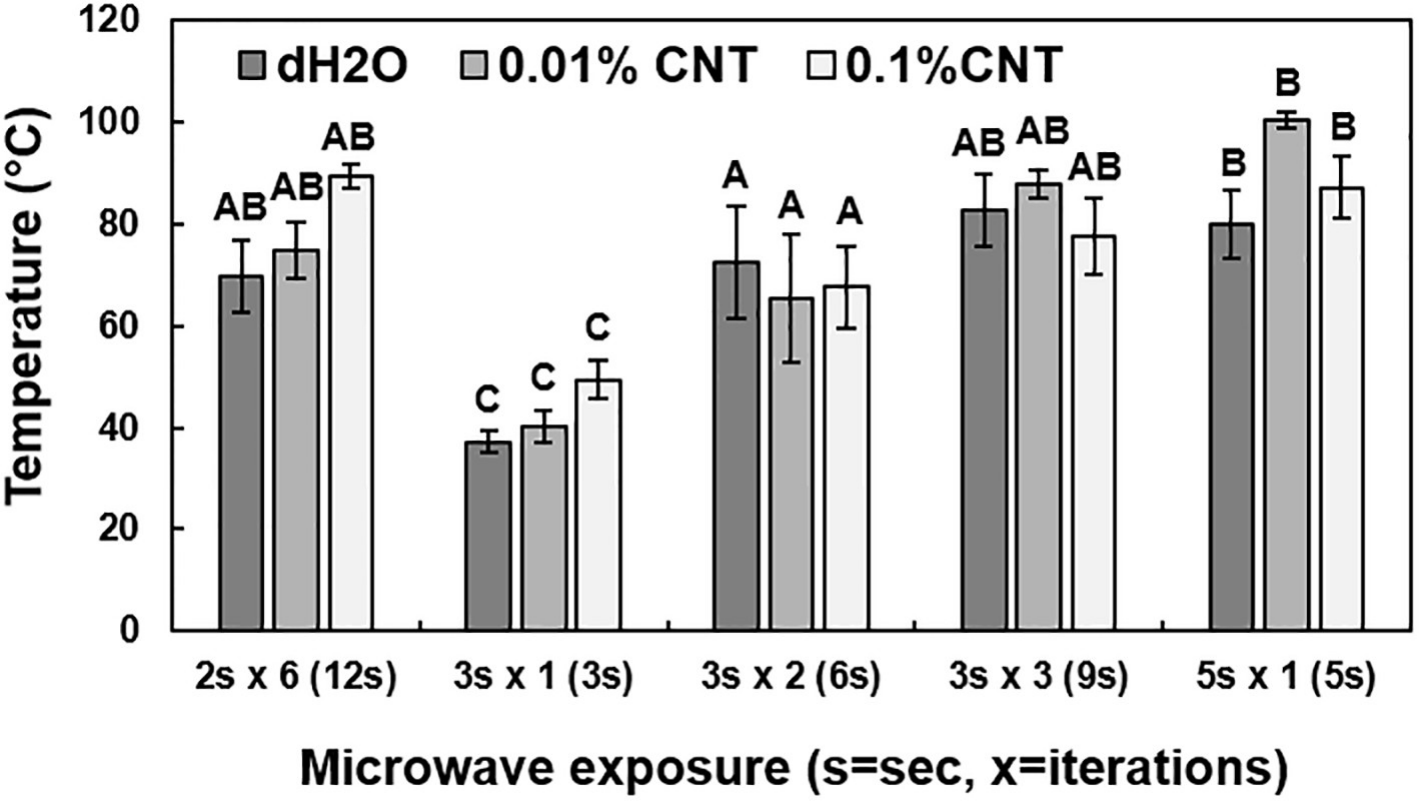
Temperature of scions following thermal treatments. Scions were first treated with sterile deionized water (dH_2_O), 0.01% (w/v) multi-walled CNTs (MWCNT), or 0.1% MWCNT dispersions and then exposed to different microwave irradiation treatments, including 2 sec in six iterations (12 sec total), 3 sec in one iteration, 3 sec in two iterations (6 sec total), 3 sec in three iterations (9 sec total), or 5 sec in one iteration. The five thermal treatments were tested to determine the optimum conditions for the remediation of *X*. *fastidiosa*. Each treatment was performed on six scion replicates. Different letters indicate significant differences.

Scions were treated with 0.1% or 0.01% (w/v) MWCNT dispersion, or dH_2_O, and then exposed to microwave radiation in a series of 5 different microwave exposure conditions, and the temperature was subsequently estimated with the thermal camera (Fig 4). The distribution of temperature measurements indicated a significant difference between the different microwave exposure conditions (*P* < 0.0001). The average temperatures following microwave radiation exposure for 3 sec or 3 sec in two iterations (6 sec total), in combination with 0.01% (w/v) MWCNT or dH_2_O as microwave absorbers, were the closest to the previously reported target temperature range (45 to 55°C) and had the lowest overall standard deviations [10]. These treatment conditions were selected for further analysis.

**Fig 5:**
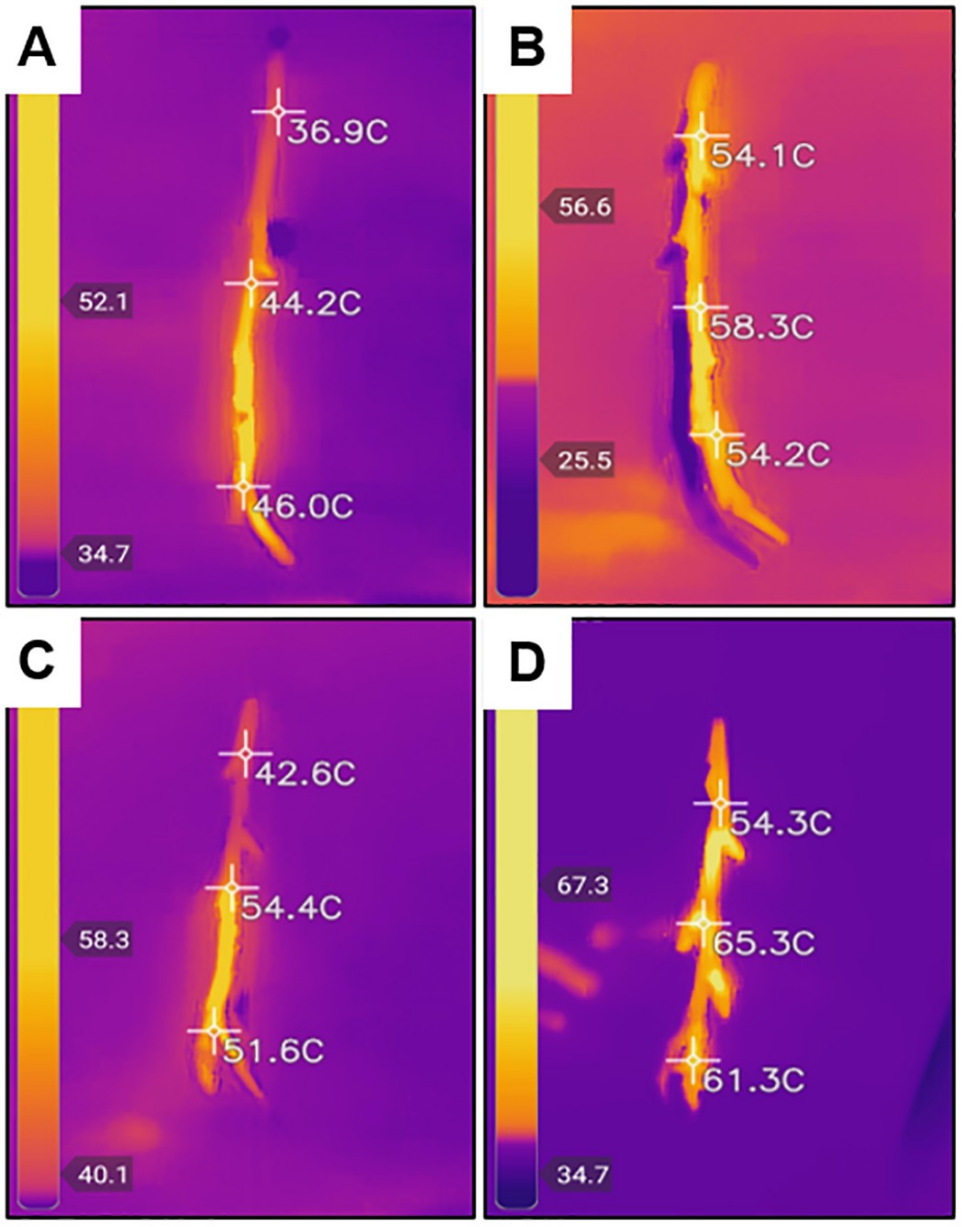
Thermal photos obtained following microwave irradiation treatment. Scion samples were first treated with sterile deionized water (dH_2_O) or 0.01% (w/v) multi-walled carbon nanotube (MWCNT) and then exposed to microwave radiation for 3 sec or 3 sec with scion readjustment in two iterations (6 sec total). A thermal camera was used to estimate 3-point temperature measurements of treated scions. (A) dH_2_O-treated and microwaved for 3 sec. (B) dH_2_O-treated and microwaved 3 sec in two iterations. (C) MWCNT-treated and microwaved for 3 sec. (D) MWCNT-treated and microwaved for 3 sec in two iterations.

**Fig 6:**
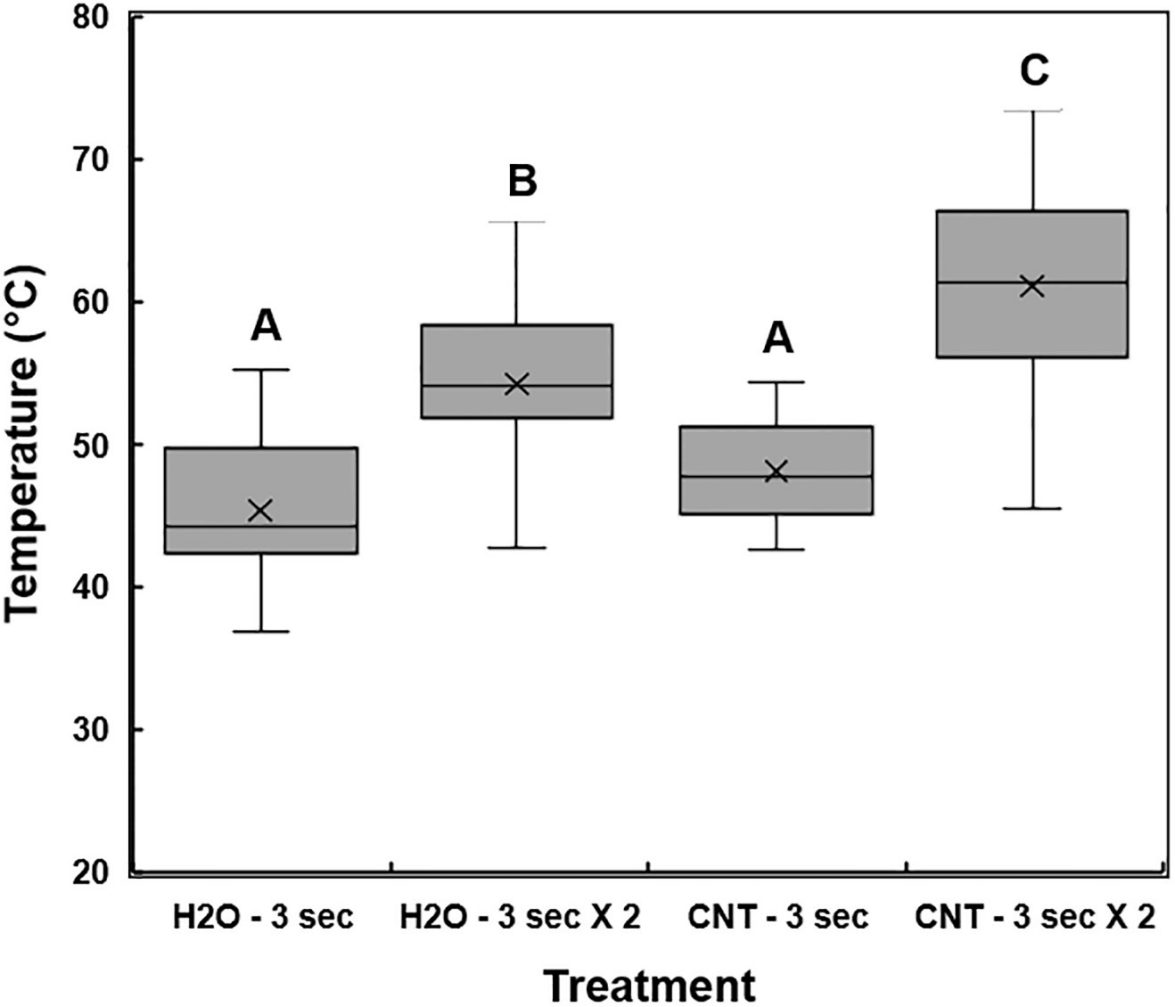
Temperature measurements in the scions following sterile deionized water (dH_2_O) or multi-walled carbon nanotube (MWCNT) treatment and microwave exposure. Scion samples were first treated with dH_2_O or MWCNT and then exposed to microwave radiation for 3 sec or 3 sec with two iterations (6 sec total). A thermal camera was used to perform 3-point measurements on the proximal, central, and distal sections of the treated scion wood. The sample treatments are plotted against the average temperature readings from each section of the scions. The 5-number summary statistic, represented as a box and whisker plot, includes the minimum, 1^st^ quartile, median, 3^rd^ quartile, and the maximum temperature values calculated for each microwave irradiation treatment. The X within each box represents the treatment mean. Different letters indicate significant differences.

The distribution of temperature measurements was compared in scions treated with dH_2_O or 0.01% (w/v) MWCNT dispersion and exposed to microwave radiation for 3 sec or 6 sec (3 sec in two iterations) (Figs 5 and 6). Significant differences in the mean temperatures of treated scions were identified between the two microwave exposure conditions (*P* < 0.0001). MWCNT-treated scions, when exposed to microwave radiation for 3 sec in two iterations (6 sec total), were significantly different when compared to dH_2_O or MWCNT treatments with microwave exposure of only 3 sec, as well as the dH_2_O-treated scions with microwave exposure for 3 sec in two iterations (*P <* 0.05). There was no significant difference in the average temperature of scions treated with dH_2_O or MWCNT and a microwave exposure duration of only 3 sec. Due to the presence of temperature readings lower than the minimum target of 45°C in the MWCNT treatment groups of microwave exposure for 3 sec, a microwave duration of 3 sec in two iterations (6 sec total) was used for all downstream analyses. Therefore, the optimum temperature for *X*. *fastidiosa* remediation was increased to a range of 55 to 65°C.

### Scion wood viability

**Fig 7:**
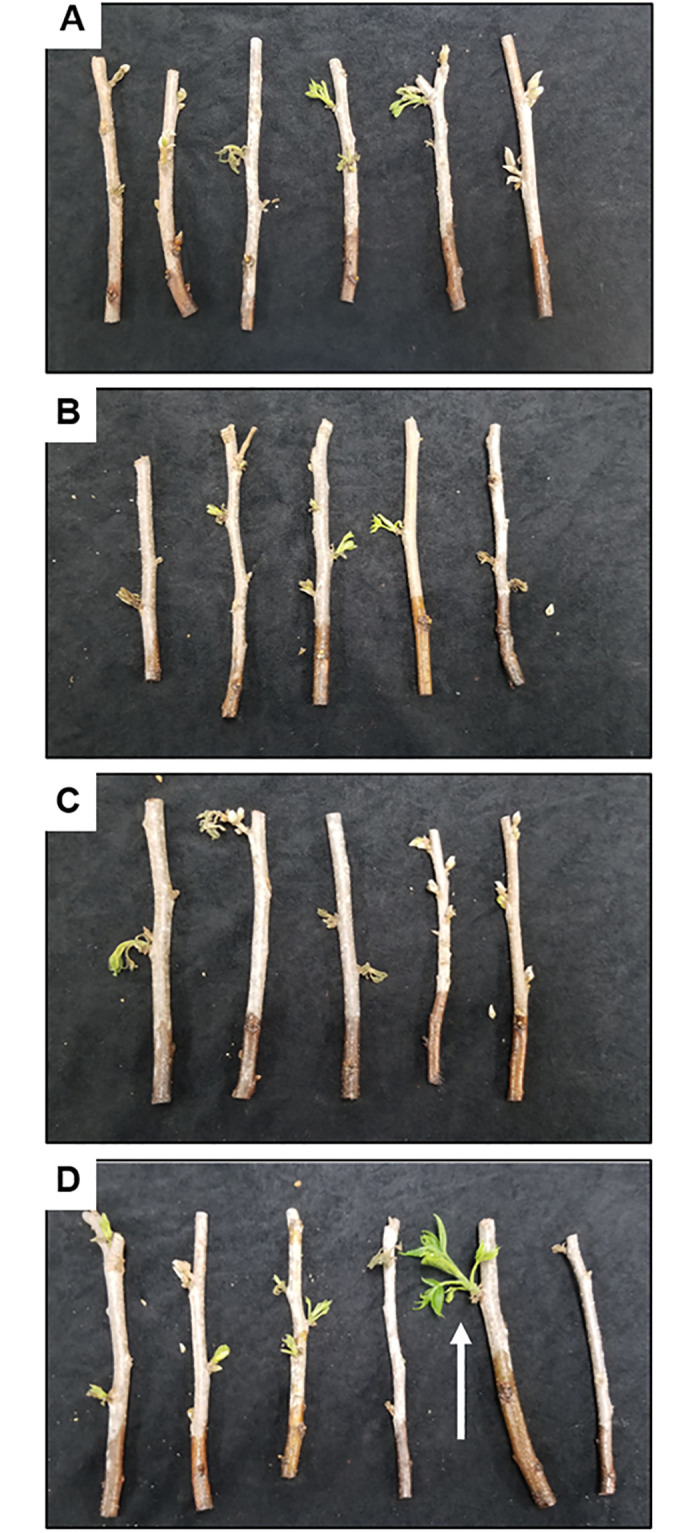
Impacts of thermal treatments on the plant viability. Scions were treated with multi-walled carbon nanotube (MWCNT) or sterile deionized water (dH_2_O) with or without microwave radiation exposure for 3 sec in two iterations (6 sec total). Scions were then incubated on an aeroponic system and monitored for two weeks. Images show the collected scions where initiation of bud-break and axillary outgrowth has occurred. (A) dH_2_O-treated scions without microwave irradiation. (B) dH_2_O-treated scions with microwave irradiation. (C) MWCNT-treated scions without microwave irradiation. (D) MWCNT-treated scions with microwave irradiation. Arrow indicates outgrowth of axillary bud.

A microwave exposure duration for 3 sec in two iterations (6 sec total) was selected for the viability assay. Percent viability was determined by the number of scions that initiated outgrowth from axillary buds or underwent bud-break per the total number of replicates treated by the following four thermal treatment conditions: 1) 0.01% (w/v) MWCNT-treated scions with microwave radiation exposure = 40.0% ± 12.2, 2) 0.01% MWCNT-treated scions without microwave = 33.3% ± 11.8, 3) dH_2_O-treated scions with microwave = 40.0% ± 12.2, and 4) dH_2_O-treated scions without microwave = 46.7% ± 12.5 (Fig 7). A chi-square analysis found no significant difference in the percent viability amongst the four treatment groups (*P* > 0.05).

### Determination of *X*. *fastidiosa* disinfection

The standard curve of the qPCR assay was robust (*R*^2^ = 0.9897) (S3 Fig). The proportion of scions that tested positive (ΔRn > 0.02, Ct < 38) for the presence of *X*. *fastidiosa* was found to be significantly different between the treatment groups by chi-square analysis (*P* = 0.0084). The percentage of *X*. *fastidiosa*-positive samples was significantly lowered by the thermal treatments with microwave for 6 sec (3 sec in two iterations). The microwave irradiation with dH_2_O microwave absorber was the most effective and comparable to the conventional HWT (Table 2). The microwave irradiation with the CNT absorber was marginally significant (*P* = 0.059) in reducing the presence of *X*. *fastidiosa* when compared to the control group (dH_2_O treatment without microwave).

**Table 2:** Detection of *Xylella fastidiosa* in pecan scions after thermal treatments with sterile deionized water (dH_2_O) or carbon nanotube (CNT) with microwave irradiation for 3 sec in two iterations compared to the controls without microwave irradiation and the conventional hot water treatment (HWT).

Treatment	dH_2_O + microwave	CNT + Microwave	dH_2_O + no microwave	CNT + no microwave	HWT 46°C water for 30 min
Percent positive for presence of *X*. *fastidiosa*	10.8 ± 4.5	15.2 ± 5.2	37.5 ± 12.1	18.7 ± 9.7	4.4 ± 3.0
*P*-value of a pairwise comparison with the control (dH_2_O + no microwave)	0.016	0.059	-	0.102	< 0.001

## Discussion

This study proves a new concept of alternative phytosanitary approaches for preparing pathogen-free pecan graftwood, which is important for reducing the risk of introduction and spread of *X*. *fastidiosa*. The novel thermal treatments based on microwave irradiation with combinations of microwave absorbers (dH_2_O and CNTs) were developed, optimized, and demonstrated to be effective in remediating *X*. *fastidiosa* contamination in pecan scions. Thermal treatments of scions by microwave radiation exposure for 6 sec (3 sec with two iterations) resulted in a lower percent positive of *X*. *fastidiosa* when compared to the control groups that were not treated with microwave irradiation. The effectiveness of disinfection by the new thermal treatments was comparable to the HWT, which had been the only practical option for sanitizing pecan scion wood before national or international distribution.

When compared to heating by conduction and convection, microwave radiation has advantages in rapid and selective heating [25]. Microwave radiation has been used to treat seed in the remediation of seed-borne pathogens, such as *Fusarium* spp. [29, 39]. This study is the first report of novel thermal treatments which combine the use of microwave absorbers and irradiation to treat plant disease in living plant graftwood without compromising the plant viability. Understanding how microwave absorbers (dH_2_O and CNTs) and microwave radiation interact within the plant substrate was critical to developing an optimal protocol that would be effective in eliminating *X*. *fastidiosa* while causing negligent thermal stress to pecan scions. Based on [10], a target temperature range of 46–55°C served as the baseline for our protocol development.

Microwave exposure times were evaluated to optimize treatment conditions. Readjustments of the scion between microwave iterations improved the direct and uniform exposure of the samples to the cavity magnetron, and reduced the variability of temperatures within the proximal, central, and distal sections of the wood. Equal distribution of heat across the scion is important to maintain the critical minimum temperature for the elimination of *X*. *fastidiosa* and to prevent dangerous temperature spikes found to damage the scions after prolonged microwave radiation exposure (S4 Fig). Ultimately, microwave irradiation in a 3-sec iteration and then readjusted before microwaving an additional 3 sec, was the most effective in achieving the optimum temperature range. At this condition, temperatures were slightly elevated so all sections of scion samples could reach the minimum target temperature of 46°C and did not show evidence of heat damage.

CNT transpiration through the xylem of scions prior to microwave exposure effectively increased temperature of scions when compared with dH_2_O as a microwave absorber. However, there was no difference found between these microwave absorbers in reducing the presence of the bacterium in scions. Microwave radiation exposure was generally successful compared with the control groups that were not treated with microwave irradiation. Further study will be needed for improving conditions for increasing the efficacy of the thermal treatments depending on microwave absorbers. Some of the factors affecting the treatment efficacy include the distribution of the absorbers throughout the xylem and heat dissipation following microwave exposure.

*X*. *fastidiosa* is slow growing and difficult to culture in vitro, which is a limiting factor in the research of this pathosystem [40, 41]. qPCR analysis also does not differentiate between viable and nonviable cells of *X*. *fastidiosa*. During the course of this research, Sicard et al. [42] published a method using qPCR in company with propidium monoazide (PMA or PMAxx) to specifically detect living cells of *X*. *fastidiosa* in vitro, but still this new method needs optimization for application in planta. Nevertheless, the likelihood for false positives due to non-living bacterial cells would represent an overestimation of the presence of *Xylella*, and does not address the more problematic scenario in which the bacterium remains in low quantities below the level of detection. The effectiveness of HWT in eliminating *X*. *fastidiosa* in this study was consistently high and equivalent to results of the previous study by Sanderlin and Melanson (2008) [10], which indicates a reasonable accuracy in qPCR detection in this study. Given the limitation of the PCR technology, the effectiveness of our thermal treatments was significant in reducing the presence of *X*. *fastidiosa* in contaminated scions, and if adopted, the new technique will supplement the current HWT and slow the spread of the bacterium through grafting.

Scion wood is typically harvested during the dormant part of the growing season prior to grafting, which follows the spring occurrence of bud-break [43]. An incubation temperature less than 9°C for CNT treatments was imperative to prevent outgrowth of axillary buds [44], which may be susceptible to damage inflicted by downstream microwave exposure. Temperatures higher than 45°C can injure young axillary shoots and leaflets, whereas buds have an increased heat capacity [45]. Furthermore, a number of chill hours are necessary for the vegetative outgrowth of pecan axillary buds to occur in the spring [46, 47]. Therefore, dormant scions kept in refrigeration after sampling and during the CNT treatment will not undergo premature bud-break and can better withstand heat stress from the later thermal treatments.

The potential benefits and disadvantages of CNT on plants have been explored [48], but the information is lacking in regards to the impacts of CNTs on woody crops, such as pecan. In certain dicotyledons, CNTs have been found to promote root growth and water uptake, and can be translocated through roots, shoots, and leaflets [48–50]. Microscopic observations of dissected pecan scion samples treated with MWCNTs provides supporting evidence that CNTs can undergo transpiration in the xylem tissue, even in the absence of adventitious roots.

Since pecan will not reproduce from seeds, propagation of pecan needs grafting. Grafting requires additional resources, including available rootstock and horticultural experience to achieve moderate success [51]. Propagation of scions on the aeroponic system was used as an alternative, short-term means for determining plant viability following the thermal treatments. There were no differences in bud-break rates between scions that underwent CNTs without microwave irradiation versus those treated with dH_2_O without microwave. This demonstrates that 0.01% (w/v) CNTs do not negatively impact the viability of pecan scion wood. Vegetative outgrowth on re-propagated scions after treatment appeared healthy and free of stress symptoms when bud-break was initiated. Although understanding the long-term effects of our thermal treatments on grafting needs further research, the impacts of the thermal treatments on graft success rates will be similar with an untreated control group and a conventional hot-water treatment.

CNT treatments on plants have been reported to have phytotoxic impacts, resulting in low seed germination or reduced biomass [20, 50]. However, the phytotoxicity is dependent on CNT dosages and host plants. There may be potential long-term negative impacts of the thermal treatments to plants such as the gene expression or mutagenesis of plants [52], but this long-term aspect remains for the future study. The pathways of CNT in the environment, as well as the potential impacts of human consumption, is also a concern when developing biotechnological applications using CNTs in agriculture [20, 53]. Although further research needed, this problem would seem unlikely, as the maximum amount of absorbed CNTs was minimal (< 2 mg per 15-cm scion), and the time to tree maturity for production is greater than 6 years [54].

*X*. *fastidiosa* has been reported to be widespread in the southern U.S. and can cause significant yield losses [5, 7, 55]. If large-scale applications can be developed for the industry, this new thermal treatment will promote the remediation of *X*. *fastidiosa* contamination in pecan graftwood. For example, portability of the magnetron would allow for in-situ treatments of infected scions with shorter treatment time where HWT is not feasible. Current HWT used as an intervention measure to eliminate *X*. *fastidiosa* in pecan and grape germplasm [10, 56] can also be complemented with the new thermal treatment options.

## Conclusions

*X*. *fastidiosa* is a quarantined pathogen that can infect a wide range of plant species [57]. Therefore, phytosanitation is critical to prevent its spread. This study provides new phytosanitary methods by the microwave irradiation to remediate *X*. *fastidiosa* from pecan scions. The new thermal applications, which utilize microwave irradiation and absorbers, were found to be successful in eliminating the bacterium in scions compared with the control groups. Furthermore, the microwave irradiation with the introduction of microwave absorbers to the plant matrix was not detrimental to the plant health. These positive results are comparable to the conventional phytosanitary method by HWT. The new thermal treatment using microwave irradiation to perform rapid and selective heating of the plant xylem tissue can be time- and cost-effective comparable to the HWT method for phytosanitation of pecan graftwood.

## Supporting information

S1 Fig The aeroponic system used for scion viability assay. Treated and untreated scions are inserted into foam grommets affixed to a lid. The bucket frame which is outfitted with a submersible recirculating pump and mist spray system. Bucket frame was filled with 15 L of dH_2_O supplemented with 1 ppm azoxystrobin.
(TIF)

S2 Fig Temperature measurements in SWCNT, MWCNT and water treated samples following microwave irradiation. Scions were treated with dH_2_O, 0.01%, 0.001%, 0.0001% (v/v) SWCNT, or 0.01%, 0.001%, 0.0001% (v/v) MWCNT, or sterile dH_2_O (H2O). Untreated and treated scions were then microwaved for 4 seconds and the temperature was measured using a thermocouple. The average temperature for each treatment is plotted against the sample treatment. Error bars indicate standard error.
(TIF)

S3 Fig Standard curve of *Xylella fastidiosa* DNA. Pure gDNA from *X*. *fastidiosa* was prepared in a dilution series (0.002, 0.008, 0.04, 0.2 and 1 ng gDNA) and amplified by qPCR. The averaged Ct values from triplicate reactions of the 5-point standard curve are plotted against the known concentrations of *X*. *fastidiosa* DNA. A logarithmic line of best-fit (indicated by the dotted line) was used to determine the regression model (*R*^2^ = 0.9897).
(TIF)

S4 Fig Impact of prolonged microwave irradiation exposure on scion wood. (A) CNTs-treated scion wood exposed to the microwave radiation for 5 sec. (B) dH_2_O-treated scion wood without the microwave irradiation.
(TIF)
