# Supplementary figures and images for: Thermal treatment using microwave irradiation for the phytosanitation of Xylella fastidiosa in pecan graftwood
Source: PLoS One. 2021 Jan 20;16(1):e0244758. doi: 10.1371/journal.pone.0244758 (PMC7816998; doi:10.1371/journal.pone.0244758)

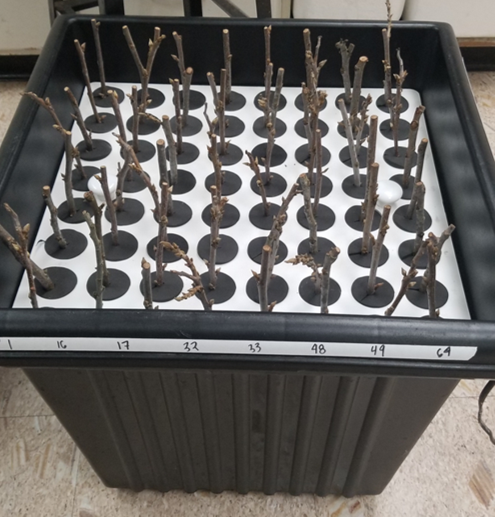

Supplement: S1 Fig — Treated and untreated scions are inserted into foam grommets affixed to a lid. The bucket frame which is outfitted with a submersible recirculating pump and mist spray system. Bucket frame was filled with 15 L of dH2O supplemented with 1 ppm azoxystrobin. (TIF) [file pone.0244758.s001.tif]

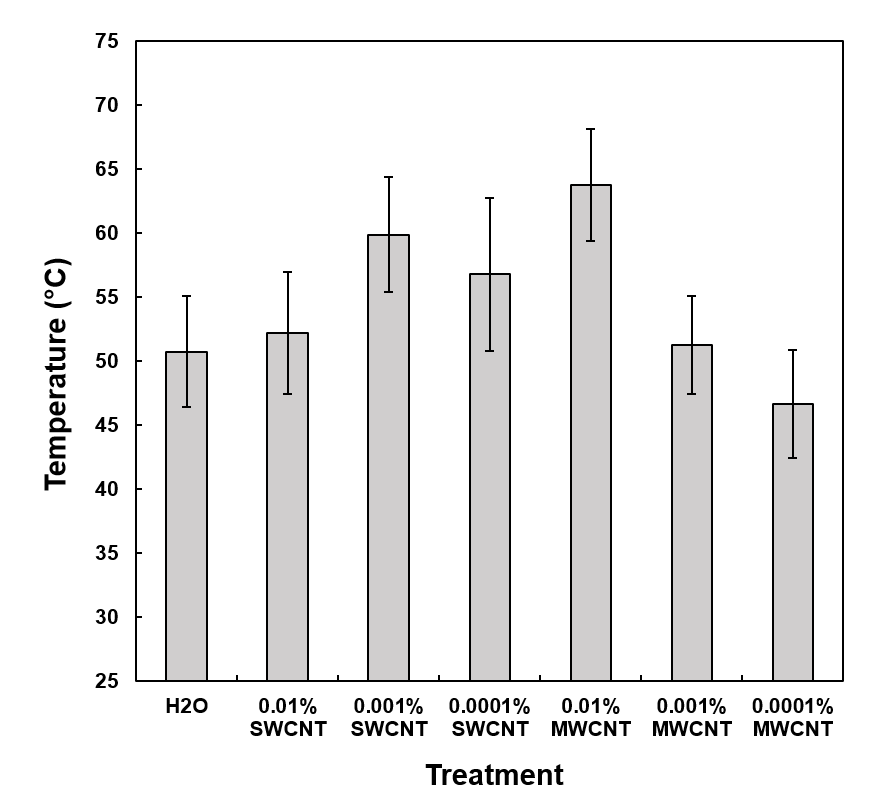

Supplement: S2 Fig — Scions were treated with dH2O, 0.01%, 0.001%, 0.0001% (v/v) SWCNT, or 0.01%, 0.001%, 0.0001% (v/v) MWCNT, or sterile dH2O (H2O). Untreated and treated scions were then microwaved for 4 seconds and the temperature was measured using a thermocouple. The average temperature for each treatment is plotted against the sample treatment. Error bars indicate standard error. (TIF) [file pone.0244758.s002.tif]

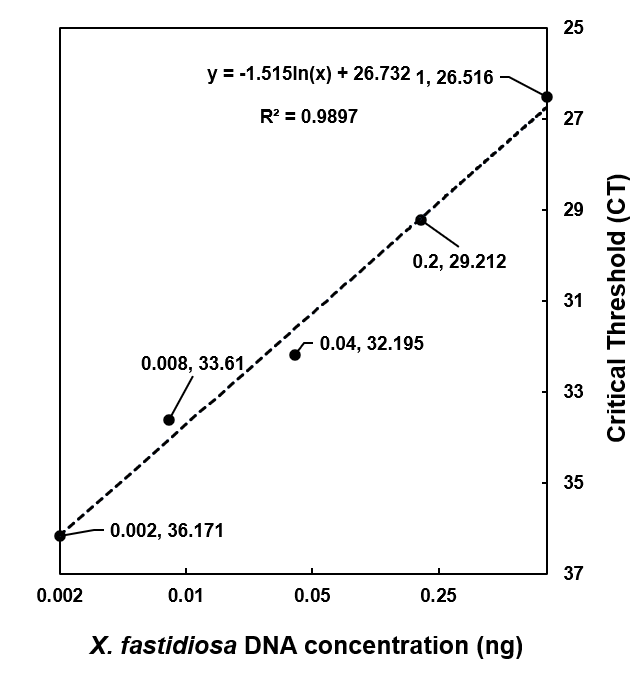

Supplement: S3 Fig — Pure gDNA from X. fastidiosa was prepared in a dilution series (0.002, 0.008, 0.04, 0.2 and 1 ng gDNA) and amplified by qPCR. The averaged Ct values from triplicate reactions of the 5-point standard curve are plotted against the known concentrations of X. fastidiosa DNA. A logarithmic line of best-fit (indicated by the dotted line) was used to determine the regression model (R2 = 0.9897). (TIF) [file pone.0244758.s003.tif]

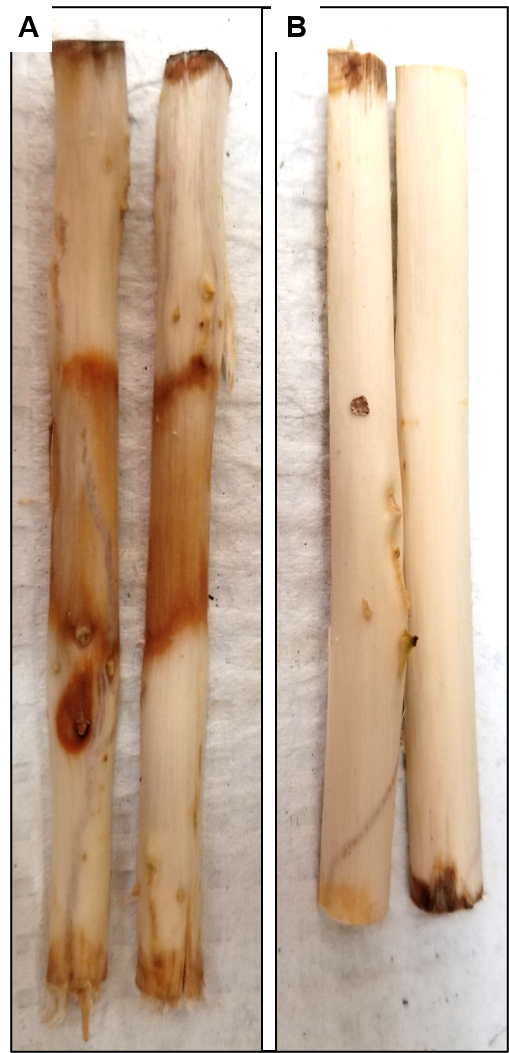

Supplement: S4 Fig — (A) CNTs-treated scion wood exposed to the microwave radiation for 5 sec. (B) dH2O-treated scion wood without the microwave irradiation. (TIF) [file pone.0244758.s004.tif]
